# Exposure to and Appeal of Tobacco Ads and Displays in China: A Qualitative Exploration of Chinese Youth Perceptions

**DOI:** 10.1093/ntr/ntad192

**Published:** 2023-10-03

**Authors:** Hannah E Barker, Lauren Czaplicki, Yuxian Cui, Rachel Shen, Qinghua Nian, Marn Xie, Joanna E Cohen

**Affiliations:** Institute for Global Tobacco Control, Department of Health, Behavior and Society, Johns Hopkins Bloomberg Schools of Public Health, Baltimore, MD, USA; Institute for Global Tobacco Control, Department of Health, Behavior and Society, Johns Hopkins Bloomberg Schools of Public Health, Baltimore, MD, USA; Institute for Global Tobacco Control, Department of Health, Behavior and Society, Johns Hopkins Bloomberg Schools of Public Health, Baltimore, MD, USA; Rising Sun Marketing Research and Consulting, Shanghai, People’s Republic of China; Institute for Global Tobacco Control, Department of Health, Behavior and Society, Johns Hopkins Bloomberg Schools of Public Health, Baltimore, MD, USA; Rising Sun Marketing Research and Consulting, Shanghai, People’s Republic of China; Institute for Global Tobacco Control, Department of Health, Behavior and Society, Johns Hopkins Bloomberg Schools of Public Health, Baltimore, MD, USA

## Abstract

**Introduction:**

Adolescents are uniquely vulnerable to nicotine addiction, and smoking is common among male adolescents in China. Although China implemented a ban on cigarette and e-cigarette advertising in public places, Chinese youth remain exposed to this marketing, which may contribute to future use.

**Aims and Methods:**

From December 2021 to January 2022, we conducted 20 online focus group discussions with 119 adolescents in 10 Chinese cities to explore sources of tobacco marketing exposure, defined as exposure to cigarette and e-cigarette ads and product displays, and what features made marketing attractive.

**Results:**

All groups discussed exposure to tobacco ads/displays in public places, including locations near their home or school. Nearly all groups discussed that exposure to online tobacco ads was common, particularly exposure to e-cigarette commercial ads and posts made by classmates or friends selling e-cigarettes. Most groups identified how eye-catching colors, imagery, product packaging, and price promotions featured in e-cigarette ads/displays attracted their attention.

**Conclusions:**

Results suggest Chinese adolescents are exposed to cigarette and e-cigarette ads and displays, many of which are placed in youth-friendly locations and contain youth-appealing features.

**Implications:**

Only a handful of studies have examined the influence of cigarette and e-cigarette advertising on youth in the context of China. Prior research has established the relationship between youth exposure to tobacco marketing and increased susceptibility to future use. Our findings emphasize the importance of effectively enforcing and expanding restrictions on cigarette and e-cigarette marketing in order to protect youth from exposure and future smoking/vaping initiation.

## Introduction

The burden of tobacco-caused death and disease in China—the world’s largest consumer of tobacco^1^—remains high,^2–4^ and rates of tobacco use among Chinese youth are cause for concern.^5^ In 2019, 5.9% of Chinese secondary school students were current smokers and 17.9% were experimental smokers.^6^ Rates of smoking are higher among boys (9.6%) compared to girls (1.9%).^6^ In addition, 45.0% of Chinese middle school students were aware of e-cigarettes and 1.2% reported using e-cigarettes, or vaping, in the last 30 days.^7^

One factor that may contribute to tobacco use among youth in China is exposure to tobacco marketing, which includes product ads and displays. There is a broad body of evidence from countries like the United States documenting the association between tobacco marketing exposure and youth tobacco use.^8,9^ Overall, exposure to tobacco ads in retail stores can increase openness to and interest in trying tobacco among youth.^10,11^ Adolescents who have been exposed to retail store and/or online tobacco ads were more likely to report initiating cigarette smoking or e-cigarette use following exposure.^12–14^ Evidence from China suggests similar patterns. A handful of studies indicate that exposure to tobacco marketing is associated with increased susceptibility to the use and use of cigarettes among Chinese adolescents.^5,6,15^

In 2015, the Chinese government implemented Article 22 of China’s Advertising Law which prohibits cigarette advertising in mass media, online, and in public places, including retail stores.^16^ As of May 1, 2022, Article 22’s advertising restrictions were extended to e-cigarettes.^17^ However, no laws are in place to ban cigarette and e-cigarette displays in retail environments in China and exposure to tobacco advertising remains commonplace, particularly among young people.^5,6^

In 2019, nearly half (46%) of junior, senior, and vocational high school students in China reported seeing tobacco advertising and promotions in retail stores in the past 30 days, and rates of exposure to marketing activities at retail stores significantly increased from 2014 (41%) to 2019 (49%).^6^ A recent study found that cigarette product displays are very prevalent near both urban and rural schools across the country, with 57% and 71% of schools having cigarette retailers within a 100 m and 250 m radius, respectively.^18^ Further, around 23% of Chinese students were exposed to tobacco advertising and promotion on the internet.^6^

These data suggest a substantial number of Chinese youth are exposed to tobacco marketing in retail stores and online, but limited information is known about the different sources (e.g. retail store type, social media platform, etc.) of marketing exposure and the extent to which young people in China find this advertising attractive. The current study fills this research gap and explores exposure to and perceptions of cigarette and e-cigarette marketing among Chinese high school students. These data can inform future marketing restrictions.

## Method

Data were collected from December 2021 to January 2022. We conducted 20 focus group discussions (FGDs) with 119 participants across 10 cities in China: Beijing, Shanghai, Chongqing, Guangzhou, Shenyang, Jinan, Kunming, Guilin, Kaifeng, and Baiyin. In each city, we conducted one FGD with girls and one FGD with boys; each FGD included 5–6 participants. All participants were in Grade 10 and between 15- and 16-years-old. Within groups, participants differed on other factors like household financial security or tobacco use status. We included only Grade 10 students because academic testing requirements were less intensive in Grade 10 versus other grades, enhancing our recruitment success. The 10 study cities differed by geographic region, population size, economic development, and administrative division (see Supplementary Table 1).^19^Table 1 shows the distribution of FGD participants by city, region, and gender.

**Table 1.** Distribution of Focus Groups by City, Geographic Region, and Gender

City	Geographic region	Girls		Boys	
		# Groups	# Participants	# Groups	# Participants
Shanghai	East	1	6	1	6
Beijing	North	1	6	1	6
Guangzhou	South	1	6	1	6
Chongqing	Southwest	1	6	1	6
Shenyang	Northeast	1	6	1	6
Jinan	East	1	5	1	6
Kunming	Southwest	1	6	1	6
Guilin	Southwest	1	6	1	6
Kaifeng	Central	1	6	1	6
Baiyin	Northwest	1	6	1	6
**TOTAL**		10 groups	59 participants	10 groups	60 participants

The Johns Hopkins Bloomberg School of Public Health Institutional Review Board (IRB No. 00017173) and Zhejiang University School of Public Health (IRB No. 0571-88208219) approved this research. Participants provided oral assent to participate and received a $25 (US) cash incentive. Parents provided oral permission for their children to participate and received a $52 (US) cash incentive. Based on consultation with research experts in China, we determined that it was more appropriate, and in line with social norms, to pay parents a higher incentive amount, while providing the young person a substantive incentive for participation.

### Sampling Approach and Eligibility

In each city, participants were purposively recruited from neighborhoods with the highest retailer density based on data from a prior observational study.^18^ A China-based organization, Rising Sun, carried out recruitment activities using three different approaches. First, Rising Sun identified and contacted any past adult (18+) research participants in their existing, proprietary database who lived in a pre-identified neighborhood and were a parent of at least one child enrolled in Grade 10. Next, they worked with existing neighborhood committees and asked for referrals to parents in the pre-identified neighborhood with at least one child in Grade 10. Finally, we used snowball sampling to recruit additional participants in Baiyin and Guilin.

Participants were eligible for this study if they were currently enrolled in Grade 10 of a senior or vocational high school; 15–16 years old; resided in a pre-identified neighborhood in one of the 10 study cities; and were able to speak and read Chinese.

### Data Collection

A team of 3 trained moderators conducted 20 virtual FGDs using Tencent, an online meeting platform software. Moderators followed the same structured discussion guide (see Supplementary Table 2) and asked questions about major sources of tobacco marketing exposure, which we defined as cigarette and e-cigarette ads and product displays, and features that influence tobacco marketing attractiveness. Participants were also shown five photos of cigarette or e-cigarette tobacco ads and displays in retail stores across China collected during a 2019 study^18^ (see Table 2). Participants ranked the photos on an attractiveness scale of 1–5 (1 = most attractive, 5 = least attractive) and discussed their rationale for the group ranking, including what features influenced attractiveness.

**Table 2.** Photos of Tobacco Ads and Displays in Retail Stores Across China that were Used in Group Ranking Activity of Ad/display Attractiveness (1 = most attractive and 5 = least attractive)

Photo name	Photo description	Photo image	Average ranking score^1^
**Q**	Electronic cigarette store inside of a shopping mall (Beijing).	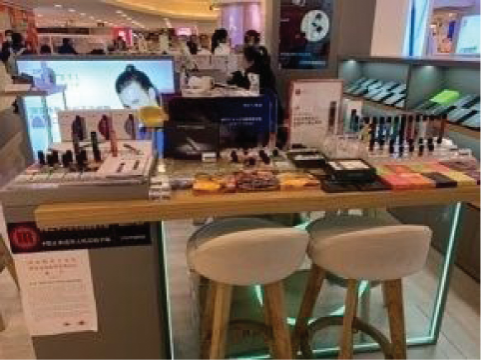	1.35 (Most attractive)
**M**	Cigarette display inside of a convenience store (Beijing).	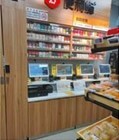	1.85
**S**	Cigarette display in a stationary store shown from the outside (Shanghai).	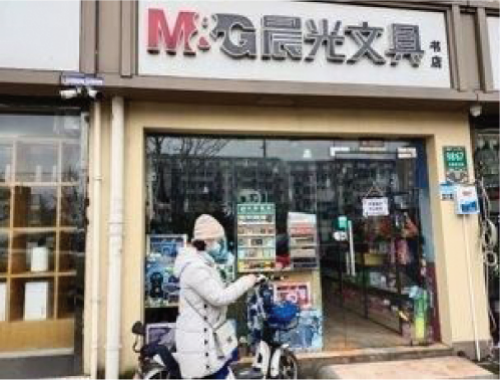	3.47
**H**	Cigarette display on a roadside stall situated outside (Kaifeng).	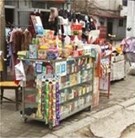	4.1
**T**	Cigarette advertisement and display shown from the outside of a tobacco retail store (Guangzhou).	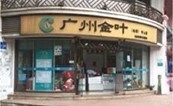	4.2 (Least attractive)

^1^Score averaged across all 20 focus groups.

After the FGD, all participants completed a short online survey. Table 3 presents descriptive statistics of participant demographics and tobacco use history.

**Table 3.** Demographic Characteristics and Tobacco Use of Focus Group Study Participants (*n* = 119)

	Girls (*n* = 59)	Boys (*n* = 60)	Total (*n* = 119)
	*n* (%)	*n* (%)	*n* (%)
**Age**			
15	23 (39.0%)	27 (45.0%)	50 (42.0%)
16	36 (61.0%)	33 (55.0%)	69 (58.0%)
**School Type**			
Senior High School	40 (67.8%)	45 (75.0%)	85 (71.4%)
Vocational High School	19 (32.2%)	15 (25.0%)	34 (28.6%)
**Financial Situation**			
Live Comfortably	17 (28.8%)	16 (26.7%)	33 (27.7%)
Meet needs with a little left	29 (49.1%)	35 (58.3%)	64 (53.8%)
Just meet basic expenses	10 (16.9%)	3 (5.0%)	13 (10.9%)
Don’t meet basic expenses	3 (5.1%)	6 (10.0%)	9 (7.6%)
**Cigarettes**			
***Current Use***			
Daily	0 (0.0%)	2 (3.3%)	2 (1.7%)
Less than daily	1 (1.7%)	4 (6.7%)	5 (4.2%)
Not at all	58 (98.3%)	54 (90.0%)	112 (94.1%)
***Ever-Use***			
Yes	8 (13.6%)	10 (16.7%)	18 (15.1%)
No	50 (84.7%)	44 (73.3%)	94 (79.0%)
*Missing, not reported*	1 (1.7%)	6 (10.0%)	7 (5.9%)
**Electronic Cigarettes**			
***Current Use***			
Daily	1 (1.7%)	3 (5.0%)	4 (3.4%)
Less than daily	0 (0.0%)	1 (1.7%)	1 (0.8%)
Not at all	58 (98.3%)	56 (93.3%)	114 (95.8%)
***Ever-Use***			
Yes	5 (8.5%)	8 (13.3%)	13 (10.9%)
No	53 (89.8%)	48 (80.0%)	101 (84.9%)
*Missing, not reported*	1 (1.7%)	4 (6.7%)	5 (4.2%)

### Coding

All FGD recordings were transcribed into Chinese and professionally translated into English. Author YC, a native Chinese and fluent English speaker, verified the quality of the translations. Three study authors (HB, LC, YC) reviewed the English transcripts for familiarity and to inform codebook development. The codebook included a priori codes (e.g. marketing exposure sources) based on the FGD guide and emerging codes from transcript review (e.g. celebrity endorsement). The coding team applied the final codebook to the same five transcripts to assess inter-rater reliability between coders. Coders met as a group to discuss and resolve any disagreements. This allowed coders to meet a high level of code-specific agreement at or above 80%. The final codebook (see Supplementary Table 3) was applied to all 20 transcripts using the qualitative software, MAXQDA 2022.1; six or seven transcripts were randomly assigned to each coder.

### Data Analysis

We conducted a thematic analysis of the coded data to identify themes regarding sources of and attractive features of tobacco marketing as defined in our study (i.e. cigarette/e-cigarette ads/displays). We also examined differences and similarities in how groups discussed themes by city and gender. Results were consistent across groups by city, and any notable differences based on the gender of the FGDs are discussed. We present results via summaries and exemplary quotations, which were edited for clarity.

## Results

### Sources of Marketing Exposure (Unaided)

#### Physical Locations

Groups discussed a wide variety of physical sources of tobacco ad and display exposure, including retail stores and kiosks in their neighborhoods or near their schools, subway stations, bus stops, and shopping malls. In general, exposure to cigarette ads was less common compared to exposure to e-cigarette ads. Many groups discussed regular exposure to e-cigarette ads in retail e-cigarette stores, supermarkets, and shopping malls.

With respect to tobacco displays, most groups discussed consistent exposure to cigarette displays in supermarkets, convenience stores, or kiosks that they visited regularly. Furthermore, groups discussed how these displays were conveniently located where they would be noticed by any customer, including young people.

Participant: Generally, tobacco and alcohol products are placed in the place that can be seen as soon as you go inside the place. They would not be placed in a more distant place or a place where we cannot see and touch.

*Guilin, boys FGD*

Most groups also discussed their exposure to e-cigarette displays in locations with high levels of foot traffic or visibility, such as display counters in shopping mall department stores. Several groups also discussed seeing youth-friendly e-cigarette displays in vending game machines and novelty or variety shops.

Participant: You can also see the displays in those shops like the M&G Shop (*a chain variety and stationary store*), where you can find everything, including planners, toys, and dolls, besides e-cigarettes … It shows several items on one shelf…the [e-cigarette] device and pod are well displayed.

*Shanghai, girls FGD*

#### Online

Nearly all groups discussed that exposure to online tobacco ads was common, particularly exposure to e-cigarette ads. Groups mentioned a variety of social media platforms and shopping websites, including WeChat Merchants, WeChat Moments, Qzone, Tik Tok and Weibo. While some groups discussed exposure to tobacco ads from a brand’s account or a blogger/influencer, most groups indicated that friends, relatives, or classmates often posted social media e-cigarette ads, which may enhance credibility or interest in the post.

Participant: People in the WeChat Moments are your buddies.…If they sell you this thing, you may first think that since they also use it, this thing may be really the same as they describe.

*Chongqing, boys FGD*

Groups generally discussed how online ads or displays for tobacco products were modern and easily accessible via cell phones to young people. Many groups discussed how online ads tapped into the current trends in youth culture, which made promoted products appear “cool” and popular. Several girls’ groups also identified how online ads and videos provided an opportunity to learn more about the features of a tobacco product, including flavor ingredients (e.g. flavor capsules in cigarettes). In some cases, girls discussed that they felt more comfortable getting information online versus asking questions in a retail store.

Finally, many groups discussed the convenience and ease with which it was possible to purchase a tobacco product online through a known vendor account.

#### Traditional Media

Exposure to television, radio, and newspaper tobacco ads was rare and only mentioned by a handful of groups who highlighted exposure to cigarette ads on television/radio in the past.

### Attractiveness of Tobacco Marketing (Unaided)

#### Color

Most groups discussed how “bright,” “striking,” and “fashionable” colors, including display lighting, made tobacco marketing more attractive. Several groups discussed how adolescents are drawn to the use of eye-catching colors, which they felt were meant to pique young people’s curiosity about the products being promoted.

Moderator: What kind of tobacco displays attract your attention the most for you?

Participant: Probably those in the mall.

Moderator: Why?

Participant: Because the design of the booths is very beautiful with many different colors, so I can’t help looking at them.

*Guangzhou, girls FGD*

#### Imagery

Several groups discussed how cigarette and e-cigarette ads were most appealing when they included attractive imagery, like models/celebrities, bright logos, fashion, and skateboarding. Product-specific images were also mentioned, including showing a variety of e-cigarette pods and flavors. One group explained how the combination of different attractive images worked together to create a stylish ad.

Participant: For example…the bright red and bright purple colors, and the very young and chic dressing. And like what they have just said, skateboarding. When they are skateboarding, they get a cigarette in their hands, and this looks trend[y] or a styl[ish].

*Beijing, boys FGD*

Additionally, a few groups also mentioned how e-cigarette video ads that included sophisticated animation or other effects dramatically increased visual attention to the product.

Participant: The last time I saw [the ad] was about half a month ago, when I browsed Tik Tok. It was…particularly high-end, very magical, with special effects, very good-looking. I didn’t know it was an e-cigarette without looking closely, and finally I found out what it was. They made that video like that 3D blockbuster.

*Kaifeng, girls FGD*

#### Text Descriptors

Several groups also discussed how the use of informative text to describe unique features of the product, including what to expect when using the product (e.g. “sweet and refreshing”) increased interest in and attractiveness of an ad. In some cases, this seemed to be an intentional tactic to reduce concern about potential harm.

Participant: For example, the [flavored cigarette] capsule. [They] may say that it is not very harmful and not as smelly as traditional cigarettes …

Moderator: Why are these [advertising messages] attractive?

Participant: So, [consumers] won’t pay attention to the harm.

*Guangzhou, boys FGD*

A handful of groups also discussed how the use of memorable slogans or quotes on product ads or displays aligned with youth culture and seemed meant to attract young people, which made the product appear more appealing.

Participant: I saw an ad at the subway station. That ad used some of the current spiritual quotes as its advertising messages. After reading them, I felt that cigarettes moved with the times. Maybe others might see it differently. But for young people, we would not forget it even after a long time of reading it.

*Jinan, boys FGD*

#### Brand

For several groups, product brands, particularly e-cigarette brands, seemed relevant in assessing a product’s quality or appeal, and boys’ groups appeared to pay more attention to brand and quality than girls. Many groups mentioned e-cigarettes by brand, including Relx, Ruike, and Yooz, and we were able to identify celebrity brand ambassadors featured in advertisements and displays.

Participant: I have seen the ads of Relx. The slogan written on them is more in line with the young people’s mind. By using the celebrities, they make it easier to recommend the products to us.

*Chongqing, boys FGD*

#### Flavors

A handful of groups discussed product flavor as an attractive feature. In general, these groups identified how good-tasting flavors, including cigarette flavor capsules and fruit or candy e-cigarettes, increased their attention to ads or displays.

Participant: It [talked] about the flavors of the e-cigarette, such as the blueberry flavor. I liked eating blueberry. I felt this flavor was very attractive. I would like to try it and buy it.

*Guilin, boys FGD*

#### Packaging

Some groups also discussed how “good-looking” packaging increased attention to the product, whereas one group discussed how these designs might be meant to attract teenagers like them. Oftentimes, packaging was mentioned with another attractive feature, like color or flavor.

Participant: It feels like they make the packages so different and so good-looking and appealing. People may think it looks very nice, and everyone wants to try what it’s like.

*Baiyin, girls FGD*

#### Price and Promotion

Many groups discussed awareness of and exposure to promotional offers included in ads or displays, particularly for e-cigarettes. Examples of promotions included reduced-price offers, in-store free trial offers, and requests to repost an online promotion in exchange for a free/reduced-price e-cigarette. Several groups discussed how free trials offered an attractive way to try new products at low to no cost to the participants.

Participant: If it says free trial, I will feel more interested. When a new store opens, there is a row of colorful cartridges or cases of e-cigarettes, with the message in big characters saying, “free trial.”

*Guilin, girls focus group*

### Aided Response to Tobacco Marketing Photos

All groups discussed that they were commonly exposed to the tobacco ads and displays featured in the photo ranking activity. Most groups identified how e-cigarette displays at shopping mall counters (Table 2, Photo Q) and cigarette displays at convenience stores (Photo M) were commonly seen in their regular routines. Photos Q and M were also ranked as the most attractive across groups, while the photos of a stationery and school supply store (Photo S), an outdoor kiosk (Photo H), and a specialty tobacco store (Photo T) were ranked as less attractive overall. Most groups discussed how the neatness and colorful aesthetic of the tobacco displays in Photos Q and M, respectively, made these displays comparatively more attractive. In addition, groups cited how the convenience of the display location in both Photos Q and M enhanced their accessibility and attractiveness. In contrast, some groups found the display of tobacco in Photo S unattractive because it was so prominently placed in a store meant for youth to purchase school supplies. Most groups also discussed how the location and accessibility of the tobacco sold at the kiosk in Photo H was appealing; however, they were suspicious of the quality of the products sold. At the same time, groups were certain that the tobacco sold in the specialty store in Photo T was high quality, but the store was less attractive because it looked like it was designed for adults and the style was not appealing or inviting to young people.

## Discussion

Results from this study suggest that some Chinese youth regularly see cigarette and e-cigarette ads and displays in physical environments and online. Our findings align with prior research^6^ and indicate that, despite laws prohibiting cigarette and e-cigarette advertising in public places and online in China, adolescents remain exposed to this marketing.

In general, groups discussed how the different sources of cigarette and e-cigarette marketing exposure they identified were in locations that were part of their regular routines (e.g. malls, convenience stores, a variety of stationary store, kiosks near school, and social media). Most of these physical environments were public places where the law stipulates that cigarette advertising is not permitted.^16^ However, other sources of marketing exposure mentioned across groups were not explicitly restricted by law. These included cigarette and e-cigarette product displays in public places, like retail stores. These findings highlight the need for extending existing advertising restrictions to tobacco displays.

Our study also documents the marketing elements that may be the most attractive to young people. Groups discussed how the bright colors used in cigarette and e-cigarette marketing were appealing and enhanced curiosity to try products. Groups also indicated how ads or displays that featured attractive or fashionable models were attractive. Youth are susceptible to tobacco marketing, like e-cigarette ads, with any level of youthful design, and multiple studies have shown youth find bold, contrasting, non-neutral colors appealing.^20–22^ Our study adds to this literature and suggests that standardizing the color of any tobacco pack to a single, dull olive color may reduce product appeal and remove the ability to use attractive imagery. Research suggests that consumers find the standard, plain color less appealing,^23,24^ which emphasizes the importance of regulating color and imagery on product packs and potentially extending these restrictions to ads.

Our study findings also suggest that product branding and packaging style contributed to adolescents’ attention to tobacco marketing. Participants, regardless of tobacco use status, recalled tobacco marketing for several cigarette and e-cigarette brands. Brands with a celebrity endorsement were particularly memorable, which is a concern as research suggests celebrity endorsements of e-cigarette brands on social media have been associated with pro-tobacco attitudes and intentions to use products.^25^ Both branding and packaging play an influential role in smoking intention,^26^ and packaging features, such as pack shape and opening style can influence product appeal and harm perceptions.^27,28^ Additional policies to restrict celebrity brand endorsements and standardize pack shape and design (e.g. required size dimensions and package openings), along with implementing a standard plain pack color, could reduce the potential appeal of these features.

Other factors such as price promotions and flavors also increased the perceived attractiveness of tobacco marketing across groups. Many groups discussed how price promotions associated with cigarette and e-cigarette ads or tobacco displays, including free trial offers, increased interest in the products. Studies suggest that offering low-cost tobacco products can lead to increased uptake of tobacco products by youth,^9,29^ indicating that policies to increase tobacco product price or reduce price-promotion and free trial offers may be effective in eliminating some of the appeal of cigarette and e-cigarette products among young people in China. In addition, several groups mentioned that they were particularly attracted to and had heightened interest in fruit or candy-flavored e-cigarettes and flavor capsule cigarettes that they saw promoted in ads or displays. Past research demonstrates youths’ preference for flavored tobacco^30,31^ and flavors may lead young people to believe the flavored product is less harmful.^31–33^ In March 2022, the Chinese government prohibited the sale of all flavored e-cigarettes besides tobacco flavor.^17^ Our findings provide evidence to support maintaining and enforcing this policy and extending it to flavored cigarettes.

Our study results have several broad policy implications for the enforcement of current cigarette and e-cigarette advertising restrictions and highlight opportunities for new approaches to limit exposure to attractive marketing. First, enhanced enforcement of current advertising restrictions in China is necessary to prevent additional exposure among adolescents. This includes increasing necessary resources (e.g. monetary, personnel, etc.) dedicated to restricting cigarette advertising in public places and in traditional media as mentioned in Article 22 of China’s Advertising Law.^16^ China’s State Tobacco Monopoly Administration formulated the administrative measures to regulate e-cigarette management in March 2022 which strengthened previous legislation by extending cigarette advertising and sales to minors restrictions to e-cigarettes.^17^ Positive steps to decrease cigarette and e-cigarette marketing should be met with stringent enforcement.

Currently, Article 59 of China’s Protection of Minors law stipulates that no tobacco should be sold around schools.^34^ The prohibition of tobacco sales around schools also applies to e-cigarettes as of March 2022.^17^ These laws conflict with the findings of the study, given all participants have been exposed to tobacco advertising as minors, and specific locations of tobacco retailers were mentioned near schools. Effective and specific prohibition of tobacco retailers near schools and a new policy to prohibit tobacco displays would prevent exposure to cigarette and e-cigarette ads and displays and protect youth.^16,18^

There are other policies and approaches to limit adolescent exposure to cigarette and e-cigarette ads and displays in China and the appeal of this marketing. Our findings suggest that tobacco specialty stores, like photo T featured in the aided discussion, maybe less appealing overall to youth. Limiting sales and displays of all tobacco products to adult-only specialty tobacco stores, without visible advertising or displays, is one pathway to reduce marketing among youth.^35^ By using this approach, it is best to mandate that all customers have their ID checked upon entry, strengthening the current approach that only allows for discretionary judgment when asking youthful-looking customers to show identification. Other policies, like plain packaging and standard shape packaging, to restrict the use of attractive colors, branding, and packaging design, could reduce the amount of exposure to youth-appealing marketing. Limits on free trials of cigarettes and e-cigarettes could also reduce youth access to and interest in trying products.^36^

### Limitations

This study is subject to several limitations. Although we utilized both unaided and aided discussion techniques to generate a comprehensive list of sources of exposure to tobacco ads and displays in FGDs, our findings may not include all possible sources of exposure. Second, our recruitment approach focused on enrolling students who were in Grade 10 and from pre-specified neighborhoods in ten specific cities. As such, our findings may not be reflective of the experience of all Chinese students. However, we recruited youth who live in areas with high tobacco outlet density, ideally capturing a group of adolescents that have the potential to be exposed to tobacco ads and displays. This targeted approach also enhanced the richness of our data. Third, our discussion guide provided definitions for and examples of “advertisements” and “displays,” however, we did not provide universal definitions for cigarettes and e-cigarettes. While some participants were very knowledgeable about these products, even naming e-cigarette brands unprompted, not all participants may have had the same level of knowledge or understanding. Fourth, we conducted our analysis English, and some nuance in the original data may have been lost. Fifth, our definition of tobacco marketing for this study was limited to ads and displays. Future research should investigate exposure to other forms of marketing such as sponsored events, free branded non-tobacco merchandise, etc. Finally, all data interpretation reflects the positionality and lens of researchers at a U.S.-based university, and no direct feedback was provided by participants through member-checking.

### Conclusion

Our results demonstrate that Chinese youth are aware of and regularly exposed to cigarette and e-cigarette ads and displays in physical environments and online. Additionally, youth are subjected to ads and displays that circumvent or violate current laws, some of which may include youth-appealing features like bright colors, price promotions, celebrity endorsements, convenient locations, and messages or claims of false product safety. These findings build on previous research that highlights the importance of preventing further youth exposure to cigarette and e-cigarette product marketing. To protect young populations, urgent changes should be made to eliminate exposure to ads and displays in public and online.

## Supplementary Material

A Contributorship Form detailing each author’s specific involvement with this content, as well as any supplementary data, are available online at https://academic.oup.com/ntr.

ntad192_suppl_Supplementary_Tables_1-3

## Data Availability

Data are available upon reasonable request.
