# Supplementary material for: Exposure to and Appeal of Tobacco Ads and Displays in China: A Qualitative Exploration of Chinese Youth Perceptions
Source: Nicotine Tob Res. 2023 Oct 3;26(4):427–34. doi: 10.1093/ntr/ntad192 (PMC10959156; doi:10.1093/ntr/ntad192)
Supplement: ntad192_suppl_Supplementary_Tables_1-3 [file ntad192_suppl_supplementary_tables_1-3.docx]

**Supplemental Table 1.** 10 study cities by geographic region, population range, annual GDP, and administrative division.

| City | Geographic Region | Population Range^a^ | 2022 Annual  GDP (RMB bn)^a^ | Administrative Division |
| --- | --- | --- | --- | --- |
| Shanghai | East | >10 million | 4,465.280 | Direct-administered municipality |
| Beijing | North | >10 million | 4,161.090 | Direct-administered municipality |
| Chongqing | Southwest | >10 million | 2,912.903 | Direct-administered municipality |
| Guangzhou | South | >10 million | 2,883.900 | Capital city of province |
| Shenyang | Northeast | 5–10 million | 769.580 | Capital city of province |
| Jinan | East | 5–10 million | 1,202.746 | Capital city of province |
| Kunming | Southwest | 5–10 million | 754.137 | Capital city of province |
| Guilin | Southwest | 5–10 million | 243.575 | Prefecture-level city |
| Kaifeng | Central | <5 million | 265.711 | Prefecture-level city |
| Baiyin | Northwest | <5 million | 63.553 | Prefecture-level city |

^a^ Source: National Bureau of Statistics of China

**Supplemental Table 2.** List of questions used in focus group discussions (n=20) conducted among girls and boys across 10 Chinese cities.

| **Unaided, open discussion** | 1. (a) How often do you see or notice advertisements for tobacco products?  - Probes: Are there certain days of the week when you see these advertisements more often? Certain times of day? For example, how often do you see them on the weekends versus traveling to and from school? Or on holidays versus normal days?   (b) How often do you notice displays for tobacco products?   - Probes: Are there certain days of the week when you see these advertisements or displays more often? Certain times of day? For example, how often do you see them on the weekends versus traveling to and from school? Or on holidays versus normal days?  1. (a) Where do you most commonly see tobacco advertisements?  - Probe: On social media, television, stores, vendor machines, radio, books, or magazines, or somewhere else?   (b) Where do you most commonly see tobacco displays?   - Probe: In stores, street vendors, kiosks, on the street or somewhere else?  1. Who do you think the tobacco advertisements and displays that we discussed are intended to attract? Is it people your age, people older than you, people younger than you?  - Follow-up: Is there anything specific about advertisements or displays that you have seen that make you think these ads or display are targeted to this group?   - Probe: use of color, use of images, use of phrasing, product flavors, placement of ads or displays in areas where X group would be exposed |
| --- | --- |
| **Aided, photo-based discussion** | Now we are going to show you five images of current tobacco advertisements and displays. Please take a few minutes to look at these images. We will then ask you some questions about them. If you refer to an individual image in your answers, please refer to it by the letter on the photo. >>*Participants view the images<<*     1. How commonplace are images like this in your neighborhood? 2. To what extent do you think young people like you are exposed to tobacco advertising and displays like those in the photos?  - Follow-up: Can you think of any examples of where some young people might be more exposed to this kind of advertising or displays? For example, are there certain times or the year where this marketing is more frequent? Or are there certain neighborhoods or locations where some young people live or go to school where they are more likely to see tobacco advertising and displays in retail stores compared to young people who live or go to school in different neighborhoods?     Now we would like to hear your thoughts about the attractiveness of these images. We are going to ask you to rank these five images in order from least to most attractive. Feel free to take some time to study the images and, without talking, make some notes on your piece of paper. In a few minutes, we will ask you to discuss as a group where you think the images belong. I will lay out the images on the screen in order from least to most attractive based on the group’s consensus, and we will then discuss the results together.     1. What makes these images more attractive? What stands out?  - Probe: color, phrases, imagery, product flavors  1. What makes these images less attractive?  - Probe: color, phrases, imagery, product flavors  1. Would anyone rank these images differently? Why? |

**Supplemental Table 3.** Codebook containing code names, descriptions, applications, and example quotes used for qualitative coding and analysis.

| **Code Name** | **Description** | **Application** | **Example(s)** |
| --- | --- | --- | --- |
| **Type of Exposure *– Administrative codes apply to all sections*** | | | |
| **Tobacco Advertisements** | Administrative code to mark explicit, specific mention of tobacco advertisement(s). | Code all discussion that refers to tobacco advertising. | “I seldom see cigarette ads.”  *Beijing, girls focus group* |
| **Tobacco Displays** | Administrative code to mark explicit, specific mention of tobacco display(s). | Code all discussion that refers to tobacco displays. | “The displays are mostly seen inside a convenience store.”  *Beijing, girls focus group* |
| **Photo Prompt** | Tag when discussion is the result of being shown imagery of tobacco advertising or tobacco displays. | Code all discussion that followed or was prompted by photo ranking activity. | “The most appealing one should be [photo] Q, because we all go to the mall often.”  *Baiyin, girls focus group* |
| **Cigarettes** | Administrative code to mark explicit, specific mention of cigarettes. | Code all discussion specific to cigarettes. | “For normal cigarettes, we have already been used them, so they don’t need to do any ads.”  *Beijing, boys focus group* |
| **E-cigarettes** | Administrative code to mark explicit, specific mention of electronic cigarettes, e-cigarettes or vapes. | Code all discussion specific to e-cigarettes, electronic cigarettes, or vapes. | “I also see the display of e-cigarettes at the convenience store. There are many pods of a variety of flavors.”  *Beijing, girls focus group* |
| **Awareness & Frequency - *Discussion of how often advertisements or displays for tobacco products are seen/noticed by youths.*** | | | |
| **Frequency** | Discussion of seeing/noticing, commenting on the familiarity or rareness, or mentioning the rate of tobacco advertisements or displays. | Apply to any discussion of to what extent participants are aware of tobacco advertisements and displays present online or in physical locations. Also apply to any discussion of the extent to which participants are familiar with/exposed to tobacco advertisements and displays online or in physical locations (either prompted by photo activity or self-prompted). Can include a specific time duration, referring to a daily, weekly, monthly, or annual basis. | “Every day, I can see them in every store.”  *Chongqing, boys focus group* |
| **Weekends** | Mention seeing or noticing tobacco advertisements or displays on weekends. | Apply to any form of tobacco advertising mentioned on Friday evening, Saturdays, or Sundays. | “I went to the mall on a weekend. There is a Relx e-cigarette’s counter.”  *Beijing, boys focus group* |
| **Weekdays or**  **School Days** | Mention seeing or noticing tobacco advertisements or displays on weekdays or school days. | Apply to any form of tobacco advertising mentioned on Mondays, Tuesdays, Wednesdays, Thursdays, or Fridays before or after school hours. | “For me, if I want to buy something at school or passing by the school, probably the convenience store around is my most convenient choice. I will probably see it every time I go to this place. Basically I will see it often in a month.”  *Beijing, girls focus group* |
| **Location - *Discussion of where tobacco advertisements or displays are seen by youths.*** | | | |
| **Physical, Near School** | Discussion of seeing or noticing tobacco advertisements or displays in a physical location inside of or near a school. | Apply to any mention of physical tobacco advertising or displays that are said to be located near a school. Can also include advertisements on school grounds. | “I am studying in a university, there are e-cigarette stores in our school, there are definitely no cigarette stores.”  *Chongqing, boys focus group* |
| **Physical, Not Near School or Unknown** | Discussion of seeing or noticing tobacco advertisements or displays in a physical location that is not referenced as near a school. | Apply to any mention of physical tobacco advertising or displays that are not mentioned in reference to being inside of or near a school. | “The last time I saw similar ads was in a supermarket. There was a glass counter in front of a supermarket, on which there were a lot of cigarettes, with various texts.”  *Baiyin, girls focus group* |
| **Online, Social Media** | Discussion of seeing or noticing tobacco advertisements or displays in an online or virtual location, such as social media (e.g., TikTok or WeChat Moments). | Apply to any mention of online tobacco advertising online including chat/message platforms, social media, videos, etc. Can apply to advertisements that were intentionally or unintentionally shared on with participants. | “Sometimes I have seen them on Tik Tok. For example, when browsing on the video app, sometimes an ad may pop out, with some ads about tobacco.”  *Baiyin, girls focus group* |
| **Traditional Media** | Discussion of seeing or hearing tobacco advertisements or displays in traditional media, such as on television or radio. | Apply to mentions of tobacco advertisements seen on television or heard on the radio. | “I used to see tobacco ads on TV, but I haven't watched much TV in the last few years.”  *Guangzhou, boys focus group* |
| **Placement of Physical Ads/Displays** | Discussion of the specific placement of tobacco advertisements and displays in specific physical locations. | Can include placement inside/within or outside a physical location (e.g., in the storefront window, at checkout area, roadside). | “If it is placed in the checkout area. After the purchase, you must check out and you will see it. Maybe you may take more glances.”  *Beijing, girls focus group* |
| **Attractiveness/Appeal - *Discussion of what makes tobacco advertising, tobacco displays, or tobacco product features more attractive/appealing. Can also apply to specific features that stand out about 5 images discussed in the aided/prompted discussion of photos.*** | | | |
| **Color** | Discussion of how colors influence perceived attractiveness or appeal of tobacco products, either specific to ads/displays or to tobacco products. | Apply to any discussion on how colors influence product appeal. | “After all, when it is displayed there, its colors give people a sense of wanting to try, especially during the holidays, when there are more students.”  *Baiyin, boys focus group* |
| **Brand** | Discussion of how the specific brand of tobacco products influences perceived attractiveness or appeal of tobacco products. | Apply to any discussion of how brand names influence product appeal. | “Basically we always buy Zhongnanhai.”  *Beijing, boys focus group* |
| **Product Flavors** | Discussion of how different product flavors of tobacco influence perceived attractiveness or appeal of the product. | Apply to any discussion of how flavors of tobacco products influence product appeal. Can include mention of specific flavors or types of flavors deemed to be appealing to young people or other audiences. | “Those ads about e-cigarette tell us that it has fruit flavor, such as apple flavor, so we want to buy it.”  *Chongqing, boys focus group* |
| **Imagery** | Discussion of how the content or style of the pictorial advertisements and/or displays influence perceived attractiveness or appeal of the product. | Apply to any discussion of the imagery of tobacco advertisements, displays, and products. Can include imagery such as icons/logos, graphic depictions of health outcomes, or depictions of the experience of users (i.e., commercial portraying smokers). | “There are characters inside the ad, who are dressed up like those people in the Republic of China period.”  *Guangzhou, boys focus group* |
| **Messages & Text Features** | Discussion of the influence of messaging, descriptive language, or text choices on tobacco advertisements, displays, or products influences perceived attractiveness or appeal. | Apply to any discussion of messages that impact the appeal or attractiveness of tobacco products, ads or displays. Can include spoken or written messaging in different forms of media (e.g., slogans) or features of text like size and font. | “I saw [a tobacco ad] recently on my phone, and I saw some new flavors from the description. And there was one point that appealed to me, ‘e-cigarette can help you quit cigarette.’ That was the point that appealed to me. It was a gimmick.”  *Chongqing, girls focus group* |
| **Packaging** | Discussion of how the packing of tobacco products influences perceived attractiveness or appeal of the product. | Apply to any discussion of the packaging of tobacco products that impacts the appeal or attractiveness of the product. Can apply to any physical feature of the packaging (size, shape, materials, etc.) or general descriptions of packaging. | “I feel that the package is very beautiful, so I will look at it more.”  *Baiyin, girls focus group* |
| **Style** | Discussion of how the quality, style, or aesthetic of tobacco advertisements, displays, or products influence perceived attractiveness or appeal of the ad or product. | Apply to any mention of the style of ads, displays or products that impacts the appeal or attractiveness to young people. Can include mention of a specific style or more feeling-based descriptors (i.e., “cool,” “beautiful,” “fancy,” or “young”). | “First of all, from the decorative style, it is a little more upscale than the traditional tobacco shop.”  *Baiyin, boys focus group* |
| **Quality** | Discussion of whether the perceived quality of tobacco products (i.e., safety, realness, trustworthiness) influences the perceived attractiveness or appeal of the product by itself or of the tobacco advertisement/display. | Apply to any mention of the perceived quality of tobacco products and the impact on youth perceptions or behavior. | “Usually you can get the authentic products from an e-cigarette store. If you buy it randomly from somewhere else, you can’t guarantee the authentic product. So, we usually do not but in a random place.”  *Guilin, boys focus group* |
| **Price & Promotions** | Discussion of how the price of tobacco products or product promotions, coupons, or free sampling/giveaways (e.g., buy 2 get one 50% off) influences perceived attractiveness or appeal of tobacco products, ads, or displays. | Apply to all mentions of price, cost, or cost-related promotions for tobacco products, including offers of free samples. Also include the impact of cost and pricing on the appeal/attractiveness of tobacco products. | “Those subscription accounts post information on the WeChat Moments. If the buyer can invite a certain number of students to buy together, he can enjoy a discount. This has quite a lot of impact.”  *Shanghai, girls focus group* |
| **Convenience** | Discussion of whether the convenience of a location (i.e., mall, store, or website) and the tobacco products that they offer influences the attractiveness or appeal of purchasing or using the product. | Apply to all mentions where location convenience or product convenience impact appeal or purchasing behavior. Can include the type of store (e.g., convenience store) or the type of location (e.g., shopping mall). | “We probably spend more time in the convenience stores in our daily life, and usually some parents go to convenience stores to buy such things because it's more convenient.”  *Baiyin, girls focus group* |
| **Endorsement** | Discussion of endorsement of tobacco products by celebrities, influencers, or well-known spokespeople impacts young people’s thoughts on or behavior related to tobacco. | Apply to any discussion of support or endorsement of tobacco use, tobacco advertisements, or tobacco policies by different types of cultural influencers. | “By using the celebrities, they make it easier to recommend the products to us.”  *Chongqing, boys focus group* |
